# Comparison of accuracy of CT parameters across chest, low-dose lung, and abdominal CT in diagnosing steatotic liver disease

**DOI:** 10.1186/s12880-025-01791-1

**Published:** 2025-07-01

**Authors:** Yan Li, Jiahao Wang, Gengyu Xu, Yixin Si, Kaiyao Huang, Yinquan Ye, Yun Peng, Yuanyuan Liu

**Affiliations:** 1 Department of Radiology, The Second Affiliated Hospital, Jiangxi Medical College, Nanchang University, Nanchang, 330006 China; 2 Jiangxi Provincial Key Laboratory of Intelligent Medical Imaging, Nanchang, 330006 China

**Keywords:** Steatotic liver disease, Low-dose computed tomography, Attenuation value, Diagnosis

## Abstract

**Objective:**

This study aims to determine the accuracy of computed tomography (CT) parameters obtained from three various scanning protocols (chest CT, low-dose lung CT, and abdominal CT) in diagnosing steatotic liver disease (SLD).

**Materials and methods:**

This retrospective study included 234 individuals who underwent chest CT, low-dose lung CT, or abdominal CT. SLD presence or absence was confirmed through ultrasound in all participants. Two radiologists independently measured the CT attenuation values of the liver (CT_L_) and spleen (CT_S_). The differences (CT_L−S_) and ratios (CT_L/S_) between liver and spleens values were calculated. Independent sample t-tests or Mann–Whitney U tests were used to compare CT_S_, CT_L_, CT_L−S_, and CT_L/S_ between SLD and control groups. One-way analysis of covariance or Kruskal–Wallis H tests were conducted to compare the parameters across scanning protocols. Receiver operating characteristic (ROC) analysis was performed.

**Results:**

The parameters (CT_L_, CT_L−S_, and CT_L/S_) were significantly lower in the SLD group than in the control group across all scanning protocols (*P* < 0.001). In the control group, significant differences in CT_S_ and CT_L_ were observed among the three scanning protocols (*P* < 0.05), while non-statistically significant differences were found for CT_L−S_ or CT_L/S_ across protocols in either group (*P* > 0.05). The ROC analysis revealed abdominal CT_L_ as the most accurate diagnostic marker for SLD (area under the curve [AUC]: 0.894, sensitivity: 90.91%, specificity: 83.33%). CT_L−S_ maintained stable diagnostic performance across protocols (AUC range: 0.813–0.816). The low-dose protocol achieved the best performance for CT_L/S_ (AUC: 0.810), demonstrating high specificity (94.34%) despite moderate sensitivity (64.81%).

**Conclusion:**

Both chest CT and low-dose CT-derived parameters demonstrated diagnostic accuracy comparable to that of abdominal CT in assessing SLD, suggesting their potential as viable alternatives in specific clinical scenarios.

## Introduction

Steatotic liver disease (SLD), a term proposed by the American Association for the Study of Liver Diseases, the European Association for the Study of the Liver, and the Asociación Latinoamericana para el Estudio del Hígado, represents a refined designation for fatty liver disease [1]. It is characterized by excessive fat accumulation in hepatocytes and has emerged as one of the most prevalent forms of chronic liver disease worldwide [2–4]. SLD encompasses various subtypes, including metabolic dysfunction-associated SLD, alcohol-related liver disease, and cryptogenic SLD. This rising incidence of disease mirrors the growing prevalence of metabolic syndrome and obesity [5]. The pathological progression of SLD spans steatosis to steatohepatitis, cirrhosis, and even hepatocellular carcinoma [6]. Moreover, SLD is closely associated with other metabolic disorders, including dyslipidemia, hypertension, and type 2 diabetes mellitus [2, 4]. Early diagnosis and management are critical for preventing these serious complications and improving patient outcomes [7].

Non-invasive imaging techniques, including ultrasound, computed tomography (CT), and magnetic resonance imaging, have become essential tools in assessing SLD. Numerous studies have demonstrated the effectiveness of these methods in detecting and evaluating SLD [8–11]. Among these techniques, CT is commonly used in clinical settings due to its high-density resolution. Specifically, decreased hepatic attenuation values (liver CT values ≤ 40 Hounsfield units [HU]) are often used as thresholds for diagnosing SLD [12, 13]. However, different scan parameters and protocols can significantly impact CT measurements [14, 15]. Moreover, the same tissue may exhibit varying attenuation coefficients under different tube peak voltages. Additionally, variations in tube current influence the signal-to-noise ratio, which can compromise the stability of CT value measurements.

In routine clinical practice, it is frequently observed that chest CT scans, including both conventional and low-dose lung scanning protocols, sometimes reveal decreased hepatic CT values. However, subsequent abdominal ultrasound or CT scans often indicate normal liver fat content, raising concerns about the diagnostic accuracy of assessments based solely on non-abdominal imaging protocols such as chest CT or low-dose lung CT. This study aims to evaluate whether non-abdominal imaging modalities, including chest CT and low-dose lung CT, can provide diagnostic accuracy for SLD compared to that of abdominal CT. By addressing this question, we seek to clarify the potential use of these imaging protocols for incidental detection of SLD.

## Materials and methods

### Study population

Medical records were reviewed to identify individuals who underwent chest CT, low-dose lung CT, or abdominal CT at our institution between June 1, 2024, and June 15, 2024. The inclusion criteria were as follows: (1) Individuals who had undergone abdominal ultrasound within seven days of the CT scan to confirm the presence or absence of SLD and (2) those with blood-based laboratory indicators of liver function. The exclusion criteria were as follows: (1) History of malignancy including liver cancer; (2) the presence of cirrhosis and trauma that could affect liver density; (3) absence of the spleen; (4) poor image quality; (5) evidence of liver dysfunction. The study was approved by the Medical Research Ethics Committee, which waived the requirement for informed consent.

A total of 271 individuals with both CT scans and abdominal ultrasound were identified from the medical records. However, 37 cases were excluded for the following reasons: 13 exhibited a history of malignancy, 5 had cirrhosis, 1 was suspected of hepatic contusion due to trauma, 1 had an absent spleen, and 14 indicated liver dysfunction. Additionally, three cases were excluded due to poor image quality. Therefore, the final study population consisted of 234 individuals who had undergone abdominal ultrasound within seven days of their CT scans. Participants were divided into the SLD group and the normal control group based on the ultrasound reports, which were issued by ultrasound physicians with at least five years of experience. The study was granted a waiver of informed consent by the Institutional Review Board following applicable ethical guidelines, given the retrospective nature of the research.

### CT scanning

All scans were performed using three CT machines: Philips IQon-Spectral CT, Philips iCT 256 CT, and UIH uCT 960+. The scanning parameters were as follows: (1) Low-dose lung CT: Tube voltage of 120 KVp, automatic tube current (10–30 mAs). (2) Chest CT: Tube voltage of 120 KVp, automatic tube current. (3) Abdominal CT: Tube voltage of 120 KVp, tube current of 250 mAs. The slice thickness was 5 mm with a 5 mm interval.

### Measurement of CT values

Two experienced radiologists, with 12 and 7 years of experience, respectively, conducted CT value measurements using a Philips workstation (Koninklijke Philips, Version 12). Measurements were based on 5-mm-thick axial images, and CT values were recorded in HU.

For each subject, measurements were taken from both the left and right lobes of the liver by delineating two circular regions of interest (ROIs) at the mid-section of the liver parenchyma. ROIs were positioned 5 mm away from the liver capsule to avoid partial volume effects while carefully excluding blood vessels, bile ducts, cysts, or other abnormalities. The liver CT value (CT_L_) for each individual was calculated as the average of the measurement from the left and right lobes. The subsequent analysis was based on the mean value obtained by both radiologists. Similarly, the spleen CT value (CTs) was determined by outlining a circular ROI at the mid-section of the spleen. The difference (CT_L−S_) and ratio (CT_L/S_) between the liver and spleen CT values were then calculated.

### Statistical analysis

All statistical analyses were conducted using Statistical Package for the Social Sciences software (version 29.0) and MedCalc 22.009, and figures were created using GraphPad Prism software (version 10.2.3). The normality of data was assessed using the Shapiro–Wilk test. Normally distributed variables are presented as mean ± standard deviation, while non-normally distributed variables are expressed as median (interquartile range). The intraclass correlation coefficient (ICC) was used to evaluate interobserver agreement for CT value measurements. The ICC values were categorized as follows: Poor (ICC < 0.2), fair (0.2 ≤ ICC ≤ 0.4), moderate (0.4 < ICC ≤ 0.6), good (0.6 < ICC ≤ 0.8), and excellent (0.8 < ICC ≤ 1.0) [16].

Independent sample t-tests were conducted to compare CT_S_ between SLD and control groups for each imaging protocol. Mann–Whitney U tests were employed to compare non-normally distributed variables, including CT_L_, CT_L−S_, and CT_L/S_. One-way analysis of variance was utilized to assess differences in CT_S_ among the three imaging protocols within SLD and control groups. Kruskal–Wallis H tests were applied for comparisons of non-normally distributed variables across imaging protocols to compare (CT_L_, CT_L−S_, and CT_L/S)_ across the three protocols. Receiver operating characteristic (ROC) curves were generated to evaluate the diagnostic performance of each protocol in identifying SLD. The area under the curve (AUC), sensitivity, specificity, and optimal cutoff values were calculated using MedCalc software. All statistical tests were two-tailed, and a *P* < 0.05 was considered statistically significant.

## Results

### Population characteristics

A total of 234 individuals who underwent abdominal ultrasound and low-does lung, chest, and abdominal CT within one week were included in this study. Among them, 107 participants underwent low-dose lung CT scans, comprising 65 males (60.7%) and 54 SLD-positive cases (50.5%). Additionally, 98 individuals underwent chest CT scans, with a mean age of 56.45 ± 13.81 years, including 61 males (62.2%) and 41 SLD-positive cases (41.8%). Furthermore, 29 participants underwent abdominal CT scans, with a mean age of 63.24 ± 10.77 years, consisting of eight males (27.6%) and 11 SLD-positive cases (37.9%). Significant differences in age and sex distribution were observed among the three groups (*P* < 0.001, F = 18.994; *P* = 0.011), while the proportion of SLD-positive cases did not differ significantly (*P* = 0.082).

### Comparison of CT parameters between groups

The ICC for CT_L_ was 0.965, and that for CT_S_ was 0.847, indicating excellent stability. The average values of two radiologists were used for additional analysis.

**Fig. 1:**
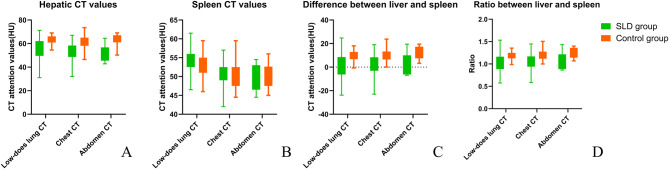
The CT parameter comparison between steatotic liver disease (SLD) and control groups. (**A**) Liver CT value; (**B**) spleen CT value; (**C**) difference between liver and spleen; (**D**) the ratio between liver and spleen

Table 1 summarizes the CT parameters (CT_L_, CT_S_, CT_L−S_, and CT_L/S_) for the three imaging protocols in both groups. For the low-dose lung CT protocol, CT_L_ was 63.75 (60.88, 66.13) in the control group and 57.63 (49.56, 62.13) in the SLD group, while CT_S_ was 53.34 ± 2.62 and 54.36 ± 2.99, respectively. For the chest CT protocol, CT_L_ was 61.00 (58.13, 65.00) in the control group and 52.75 (48.63, 58.38) in the SLD group, while CT_S_ was 50.68 ± 3.52 and 50.82 ± 3.34, respectively. For the abdominal CT protocol, CT_L_ was 63.50 (61.00, 67.06) in the control group and 51.00 (45.50, 56.75) in the SLD group, while CT_S_ was 50.22 ± 3.31 and 49.73 ± 3.62, respectively. Regardless of the scanning protocol, statistically non-significant differences in CT_S_ were observed (*P* > 0.05), while CT_L_, CT_L-S_, and CT_L/S_ in the SLD group were lower than those in the control group (*P* < 0.001; Fig. 1).

**Table 1:** CT parameters of the SLD group and control group under different scanning protocols

	Low-dose lung CT (*n* = 107)			Chest CT (*n* = 98)			Abdominal CT (*n* = 29)		
	Control (*n* = 53)	SLD (*n* = 54)	*P* value	Control (*n* = 57)	SLD (*n* = 41)	*P* value	Control (*n* = 18)	SLD (*n* = 11)	*P* value
CT_L_	63.75 (60.88, 66.13)	57.63 (49.56, 62.13)	< 0.001	61.00 (58.13, 65.00)	52.75 (48.63, 58.38)	< 0.001	63.50 (61.00, 67.06)	51.00 (45.50, 56.75)	< 0.001
CT_S_	53.34 ± 2.62	54.36 ± 2.99	0.063	50.68 ± 3.52	50.82 ± 3.34	0.841	50.22 ± 3.31	49.73 ± 3.62	0.709
CT_L−S_	9.75 (6.75, 12.88)	3.00 (–6.25, 8.56)	< 0.001	10.75 (6.50, 13.25)	2.00 (–3.13, 8.38)	< 0.001	14.13 (7.50, 17.38)	–2.00 (–6.25, 10.00)	0.005
CT_L/S_	1.18 (1.13, 1.25)	1.06 (0.88, 1.16)	< 0.001	1.22 (1.19, 1.28)	1.04 (0.94, 1.17)	< 0.001	1.28 (1.14, 1.36)	0.96 (0.89, 1.22)	0.008

*SLD, Steatotic liver disease; CT_L_, Liver CT value; CT_S_, Spleen CT value; CT_L − S_. Difference between liver and spleen; CT_L/S_. The ratio between liver and spleen

### Comparison of CT parameters among imaging protocols

The analysis revealed significant differences in CT_S_ among the three imaging protocols for both the control group (F = 12.204, *P* < 0.001) and the SLD group (F = 18.994, *P* < 0.001). Additionally, significant variations were identified in CT_L_ among the protocols within the control group (*P* = 0.037), whereas non-statistically significant differences were found for the SLD group (*P* = 0.087).

**Fig. 2:**
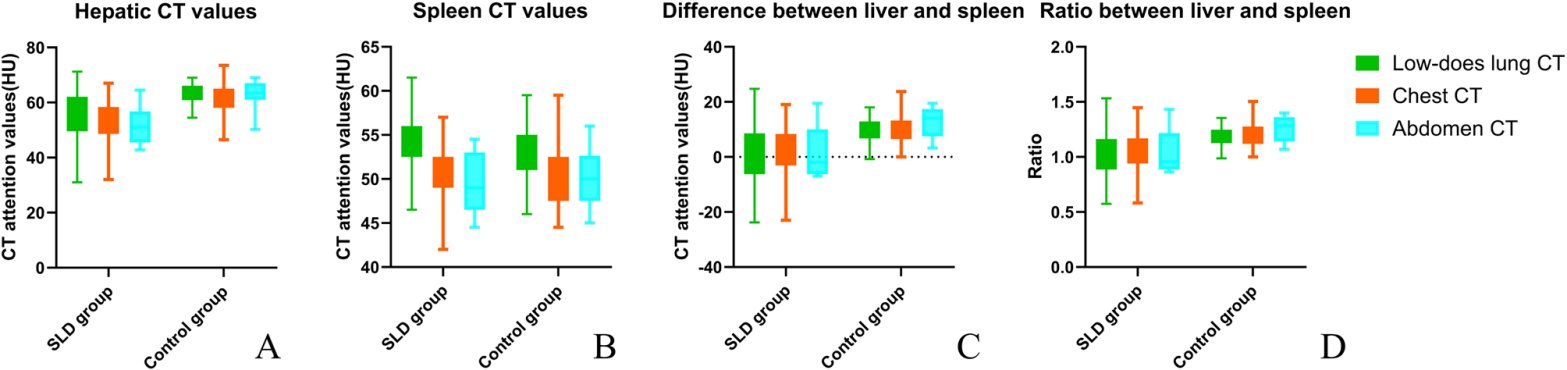
Parameter comparison between steatotic liver disease (SLD) and control groups under different imaging protocols. (**A**) Liver CT value; (**B**) spleen CT value; (**C**) difference between liver and spleen; (**D**) the ratio between liver and spleen

In contrast, non-statistically significant differences were observed for CT_L−S_ among the imaging protocols in either the control group (*P* = 0.195) or the SLD group (*P* = 0.887). Similarly, non-statistically significant variations were detected for CT_L/S_ across the imaging protocols in both groups (*P* = 0.094, *P* = 0.843). A detailed comparison of the CT parameters for each protocol is presented in Fig. 2, which summarizes the results for control and SLD groups.

### ROC analysis

Table 2 summarizes the results of the ROC analysis for different parameters (CT_L_, CT_L−S_, and CT_L/S_) across three imaging protocols (low-dose, chest, and abdomen) in the diagnosis of SLD. All AUC values for CT parameters were statistically significant (*P* < 0.01). For CT_L_, the abdominal protocol yielded the highest diagnostic accuracy (AUC: 0.894), followed by the chest (AUC: 0.825) and low-dose lung protocols (AUC: 0.793). While the abdominal protocol achieved the highest sensitivity (90.91%), it indicated the lowest specificity (83.33%). CT_L−S_ exhibited comparable AUC values across all protocols (range: 0.813–0.816), with abdominal scanning demonstrating the highest specificity (94.44%). For CT_L/S_, the low-dose lung protocol indicated the highest AUC value (AUC: 0.810), with a sensitivity of 64.81% and specificity of 94.34%, followed by the chest and abdominal protocols, which achieved AUC values of 0.806 and 0.798, respectively.

**Table 2:** The results of ROC analysis in steatotic liver disease diagnosis

Parameter	Protocol	AUC	Sensitivity (%)	Specificity (%)	Cutoff value (HU)	*P* value
CT_L_	Low-dose	0.793 (0.703–0.865)	50.00	98.11	≤ 56.5	< 0.0001
CT_L_	Chest	0.825 (0.736–0.895)	65.85	85.96	≤ 56	< 0.001
CT_L_	Abdominal	0.894 (0.723–0.977)	90.91	83.33	≤ 60	< 0.0001
CT_L−S_	Low-dose	0.813 (0.726–0.882)	61.11	94.34	≤ 5.25	< 0.0001
CT_L−S_	Chest	0.815 (0.724–0.886)	63.41	85.96	≤ 4	< 0.0001
CT_L−S_	Abdominal	0.816 (0.628–0.934)	72.73	94.44	≤ 4.75	0.0019
CT_L/S_	Low-dose	0.810 (0.723–0.879)	64.81	94.34	≤ 1.10	< 0.0001
CT_L/S_	Chest	0.806 (0.713–0.879)	63.41	85.96	≤ 1.08	< 0.0001
CT_L/S_	Abdominal	0.798 (0.608–0.923)	72.73	94.44	≤ 1.09	0.0059

* CT_L_, Liver CT value; CT_S_, Spleen CT value; CT_L−S_. Difference between liver and spleen; CT_L/S_. The ratio between liver and spleen

## Discussion

The imaging coverage range of chest CT and low-dose lung CT scans extends from the thoracic inlet to the base of the lungs, often encompassing the liver and spleen. Incidental abdominal abnormalities are frequently observed with these protocols. However, different scanning protocols produce images with varying focal points and quantitative CT values. In this study, we quantitatively assessed liver CT parameters using non-dedicated or abdominal-focused protocols and evaluated their utility in diagnosing general SLD. Our results indicated that CT_L_, CT_L−S,_ and CT_L/S_ in the SLD group were consistently lower than those in the control group across all protocols. The ROC analysis revealed that although CT_L_ from abdominal CT obtained the highest AUC for SLD diagnosis, both chest CT and low-dose CT demonstrated distinct advantages in specific metrics.

CT_L_, CT_L−S,_ and CT_L/S_ in the SLD group were consistently lower than those in the control group, regardless of the scanning protocol used. By contrast, CT_S_ was comparable in the two groups. These observations align with our initial hypotheses. Yuka et al. found that the liver CT values obtained from chest CT of psoriasis patients who are at high risk of SLD were notably lower than those in the control group. This finding suggests the potential utility of chest CT parameters in identifying steatosis [17]. However, their study did not examine how different protocols might influence CT values or assess the diagnostic accuracy of these measurements.

Our investigation determined the impact of scanning protocols on CT values. Significant differences were observed in CT_S_ values across the three protocols within SLD and control groups. Furthermore, notable variations in CT_L_ values were found among the three protocols, specifically within the control group. These results suggest that CT values are sensitive to changes in scanning parameters. This is supported by previous research, which indicates that CT values can vary depending on tube voltage when diagnosing adrenal adenomas [18] and that vertebral bone density measurement decreases as tube voltage increases [19]. Despite these differences, the absolute variations among protocols in our study were relatively small, likely due to the consistent use of a 120 KVp tube voltage. Furthermore, non-statistically significant differences were observed in the CT_L−S_ or CT_L/S_ values across the three protocols in either the control or SLD groups. This suggests that, while multiple factors may influence CT_L_ measurements, the differences and ratio parameters derived by incorporating spleen values (CT_L−S_ and CT_L/S_) may offer a more stable basis for evaluation.

Regarding the ROC analysis, while abdominal CT demonstrated superior overall performance with the highest AUC for CT_L_ parameters, both chest CT and low-dose CT indicated distinct advantages in specific metrics. Under the chest protocol, CT_L_, CT_L−S_, and CT_L/S_ demonstrated relatively strong stability performance in identifying SLD. Low-dose CT exhibited higher specificity but lower sensitivity for SLD detection; however, when incorporating CT_S_ to calculate CT_L−S_ and CT_L/S_, both sensitivity and AUC improved. This unexpected finding verifies that chest and low-dose protocols, despite not being abdominal-specific, can still yield CT parameters comparable to those from abdominal CT for diagnosing SLD. Our study aligns with previous research highlighting the diagnostic utility of low-dose CT for SLD [20] but with a key difference. We used ultrasound as the reference standard rather than positron emission tomography coronary calcium CT, and we compared three distinct protocols. The high specificity exhibited by chest CT and low-dose lung CT parameters implies that incidental SLD detections on these modalities might already represent true-positive cases.

Ultrasound was selected as the reference standard due to the rarity of patients undergoing both CT and MR proton density fat fraction (PDFF) imaging simultaneously. During the analysis, a discrepancy was observed between diagnostic results. Some individuals diagnosed with SLD by ultrasound were not identified as positive on the corresponding CT scans, while most CT-diagnosed SLD cases were confirmed as positive on ultrasound. This discrepancy may stem from several factors. First, radiologists interpreting chest or lung CT scans may focus primarily on the target region, potentially overlooking incidental abdominal findings. Second, differences in imaging techniques and diagnostic thresholds across modalities could contribute to the variability. Notably, the high specificity of chest CT and low-dose lung CT parameters suggests that incidental detections of SLD on these may already indicate true positive cases. Additionally, it is crucial to recognize that the accuracy of ultrasound in diagnosing SLD. Compared to PDFF, ultrasound exhibits lower sensitivity, which inevitably affects diagnostic accuracy [21]. Although the positive and negative cases of SLD in this study were determined by experienced ultrasonography specialists, the findings should be regarded as preliminary. Further validation using PDFF as the reference standard is necessary to confirm these results.

This study has several limitations. First, the absence of histopathological confirmation for SLD represents a significant constraint, as ultrasound serves only as an indirect means of assessing liver fat content. The findings of this preliminary study require further validation using PDFF measurements. Second, our retrospective comparison across different scanning protocols inherently includes biological differences between patient populations (particularly in liver composition and SLD pathology). Because retrospective study designs inherently limit control over imaging protocols and ethical concerns preclude unnecessary radiation exposure, we compared CT values across patient cohorts scanned with different protocols rather than prospectively assessing the same individuals with multiple CT scans. Third, a more detailed exploration of SLD etiology and its associations with comorbidities such as obesity and diabetes could further highlight the utility of CT values in assessing SLD. However, since most participants were outpatients undergoing imaging for pulmonary nodules, critical data such as diabetes history or body measurements (height/weight) often lacks completeness, limiting comprehensive investigation into SLD’s etiology and influencing factors. Forth, inter-scanner variability exists because different CT systems were used, which may potentially affect image quality and measurement consistency. Additionally, the small sample size of the abdominal CT subgroup (*n* = 29) limits statistical power and may restrict the ability to detect significant findings.

## Conclusion

In conclusion, this study analyzed liver CT parameters obtained under various scanning protocols for the diagnosis of SLD. The findings revealed that parameters derived from chest CT and low-dose lung CT scans could effectively distinguish between SLD and control groups. Although abdominal CT_L_ yielded the highest AUC, when considering the multitude of factors influencing CT values, the difference and ratio obtained in combination with the spleen appeared more stable in the evaluation. Furthermore, the AUCs of CT liver-spleen difference (CT_L−S_) and CT liver-spleen ratio (CT_L/S_) from non-dedicated abdominal CT protocols were comparable to those of abdominal CT. Future research should aim to validate results using histopathology or advanced imaging techniques such as PDFF and to collect more comprehensive clinical data to explore the etiologies of SLD and the impact of comorbidities, such as diabetes, on liver density.

## Data Availability

The data used to support the findings of this study are available from the corresponding author at [18970010699@163.com] upon request.

## Abbreviations

SLD: Steatotic liver disease
CT: Computed tomography
HU: Hounsfield units
ROI: Region of interest
CT_L_: Liver CT value
CT_S_: Spleen CT value
CT_L−S_: Difference between the liver and spleen CT value
CT_L/S_: Ratio between the liver and spleen CT value
IQR: Interquartile range
ICC: Intraclass correlation coefficient
ANOVA: Analysis of variance
ROC: Receiver operating characteristic
AUC: Area under the curve
MRI: Magnetic resonance imaging
HU: Hounsfield units
ROI: Region of interest
ICC: Intraclass correlation coefficient
